# Predictive value of pretreatment lymphocyte count in stage II colorectal cancer and in high-risk patients treated with adjuvant chemotherapy

**DOI:** 10.18632/oncotarget.5835

**Published:** 2015-10-20

**Authors:** Lei Liang, Ji Zhu, Huixun Jia, Liyong Huang, Dawei Li, Qingguo Li, Xinxiang Li

**Affiliations:** ^1^ Department of Colorectal Surgery, Fudan University Shanghai Cancer Center, Shanghai, People's Republic of China; ^2^ Department of Oncology, Shanghai Medical College, Fudan University, Shanghai, People's Republic of China; ^3^ Department of Radiation Oncology, Fudan University Shanghai Cancer Center, Shanghai, People's Republic of China

**Keywords:** pretreatment lymphocyte count, stage II colorectal cancer, prognosis, high risk, adjuvant chemotherapy

## Abstract

Pretreatment lymphocyte count (LC) has been associated with prognosis and chemotherapy response in several cancers. The predictive value of LC for stage II colorectal cancer (CRC) and for high-risk patients treated with adjuvant chemotherapy (AC) has not been determined. A retrospective review of prospectively collected data from 1332 consecutive stage II CRC patients who underwent curative tumor resection was conducted. A pretreatment LC value <1.3 Giga/L(28.1%, 373/1332) was defined as low LC. A total of 738 patients (55.4%) were considered high-risk, 459 (62.2%) of whom received AC. Patients with low LCs had significantly worse 5-year OS (74.6% vs. 90.2%, *p* < 0.001) and DFS (61.3% vs. 84.6%, *p* < 0.001). High-risk patients with low LCs had the poorest DFS (*p* < 0.001). Multivariate analysis indicated that low LC value or combined with high-risk status were both independent prognostic factors(*p* <0.001). High-risk, AC-treated patients with high LCs had significantly longer DFS than untreated patients (HR, 0.594; 95% CI, 0.364–0.970; *p* = 0.035). There was no difference or trend for DFS or OS in patients with low LCs, regardless of the use of AC (DFS, *p* = 0.692; OS, *p* = 0.522). Low LC was also independently associated with poorer DFS in high-risk, AC-treated patients (HR, 1.885; 95% CI, 1.112–3.196; *p* = 0.019). CONCLUSIONS: Pretreatment LC is an independent prognostic factor for survival in stage II CRC. Furthermore, pretreatment LC reliably predicts chemotherapeutic efficacy in high-risk patients with stage II CRC.

## INTRODUCTION

Colorectal cancer (CRC) is the third most common tumor in men, the second most common tumor in women, and the fourth leading cause of cancer-related death worldwide [1]. For stage II disease, which represents 30–40% of all resected CRCs, the five-year relative survival rate is 75%, indicating that 25% of patients relapse and die of their cancer within 5 years of surgery [2].

Several clinical and pathological features, including T4 stage, bowel perforation or clinical bowel obstruction, inadequate lymph node sampling, poorly differentiated histology, lympho-vascular and perineural invasion, have been defined as high-risk factors associated with a worse prognosis for stage II CRC [3]. Current clinical guidelines recommend adjuvant chemotherapy (AC) for such patients to prevent tumor recurrence after curative surgery [4, 5]. Although these high-risk factors do not reliably predict chemotherapeutic outcome, their presentation is typically associated with a poor prognosis. Therefore, high-risk patients possess the greatest relative benefit from adjuvant treatment [6].

However, the outcome of adjuvant treatment for these patients remains controversial. The MOSAIC trial documented a nonsignificant trend toward improved survival in patients with high-risk stage II disease who were treated with adjuvant treatment [7]. Conversely, an analysis of Surveillance, Epidemiology, and End Results (SEER)-Medicare data demonstrated a lack of survival benefit from AC in the same population [8]. Therefore, there is great interest in the elucidation of additional prognostic and predictive biomarkers that can improve outcome through patient classification.

Recent findings have revealed that cancer patient outcomes are not only determined by tumor characteristics but also by patient-related factors. Analysis of the local tumor environment has revealed the role of the immune system in preventing tumor recurrence [9]. Recent studies have reinforced the belief that defective functioning or decreased numbers of lymphocytes reduce the ability of a patient's immune system to mount an effective response to cancer cells. Pretreatment lymphocyte count (LC) is considered a surrogate marker for the level of immunosuppression in patients and has been associated with prognosis in several cancers, including hematological malignancy, breast cancer, and renal cell cancer [10–12]. It has also been suggested that low LC is associated with poor response to chemotherapy or radiotherapy [13, 14], suggesting the important role of LC in survival and clinical treatment response. Therefore, we speculated that pretreatment LC might have important predictive value in the prognosis of stage II CRC and the outcome of high-risk patients treated with AC. To our knowledge, no studies have investigated the value of LC in such patients. Therefore, we conducted a large-scale retrospective cohort study to investigate the prognostic and predictive value of pretreatment LC as a widely available marker for stage II CRC and to identify the stage II CRC patients who will best benefit from the use of AC.

## RESULTS

### Patient characteristics

**Figure 1:**
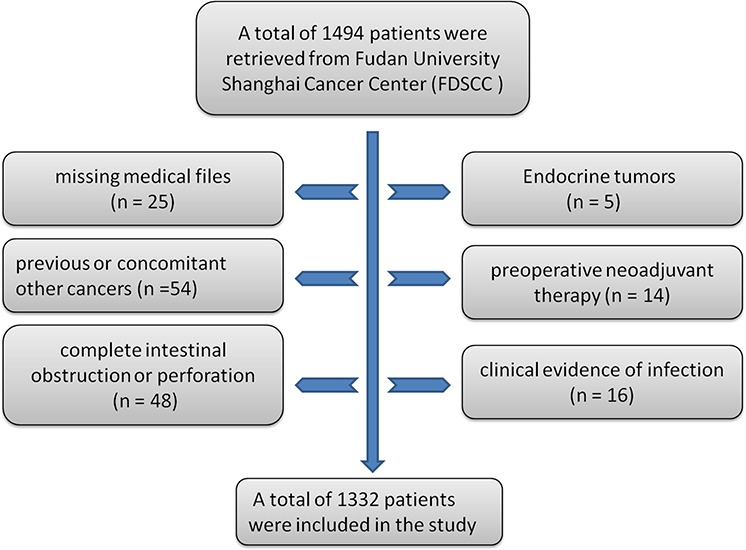
Flow chart of patients excluded from the analysis

A total of 1494 patients were retrieved from the database. Of these, 162 were excluded from the study for the following reasons: previous or concomitant other cancers (*n* = 54); complete intestinal obstruction or perforation (*n* = 48); clinical evidence of infection (*n* = 16); preoperative neoadjuvant therapy (*n* = 14); endocrine tumors (*n* = 5); and missing or inaccessible medical files (*n* = 25). Thus, 1332 consecutive patients with stage II CRC were selected for this study. Patients excluded from the analysis were shown in a flow chart (Fig. 1).

**Figure 2:**
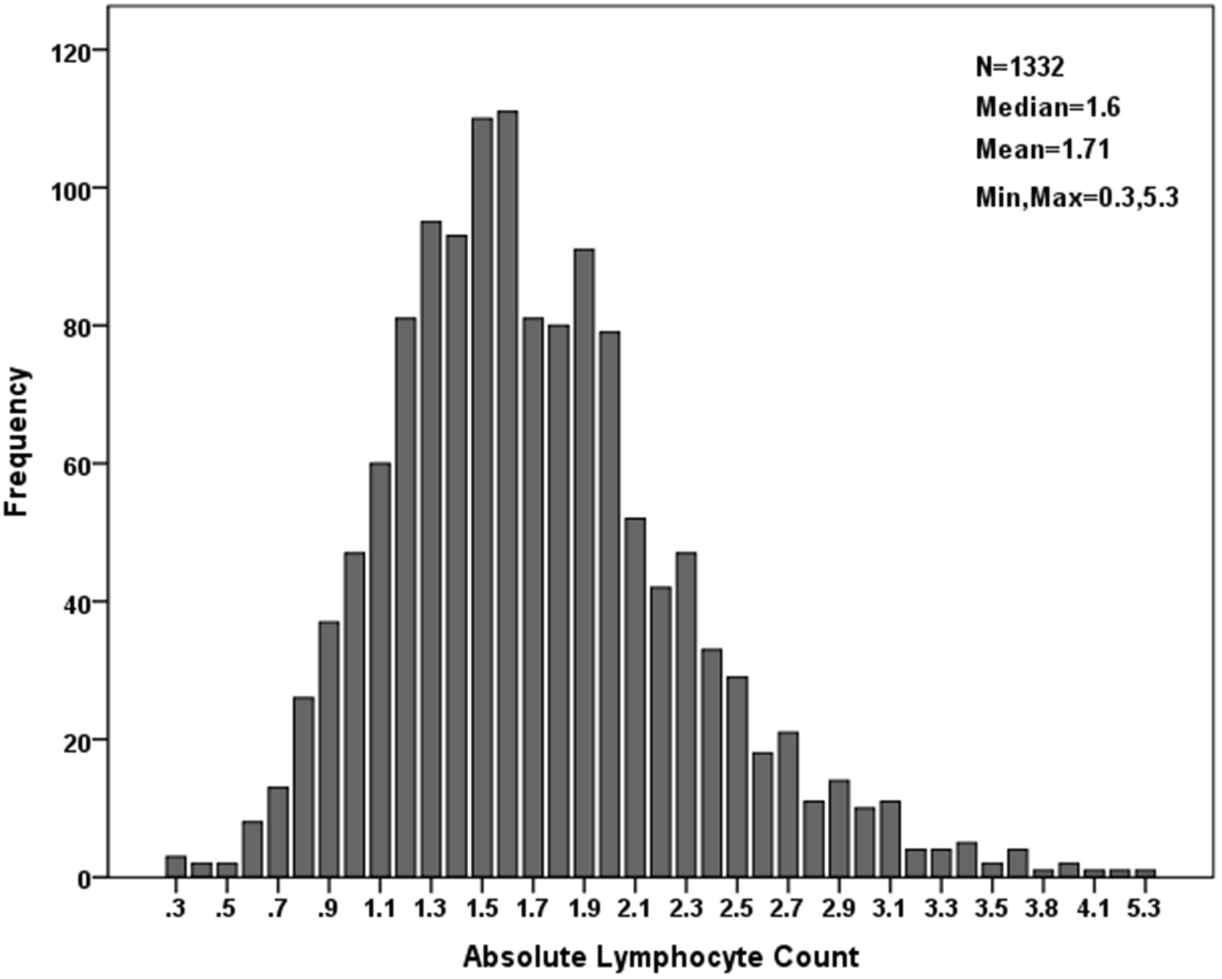
LC distribution within the cohort

Of the 1332 patients, 511 were women (38.4%) and 821 were men (61.6%). The median age for the entire cohort was 60 years (mean, 60.0; range, 17–90). The anatomic locations of the primary tumors were the colon in 688 cases (51.7%) and the rectum in 644 cases (48.3%). The median LC value was 1.6 Giga/L (mean, 1.71; range, 0.3–5.3). The LC distribution in our patients is shown in Fig. 2. The median follow-up time was 816 days (mean, 924.9 days; range, 8–2480 days). Among the patients, 738 (55.4%) were considered high-risk and 594 (44.6%) were deemed low-risk. Within the high-risk group, the most frequent poor prognostic features included T4 tumor (58.3%), suboptimal lymph node sampling (27.4%), perineural invasion (21.5%), lymphovascular invasion (13.0%) and poor differentiation (21.1%). Approximately 188 patients (25.5%) had >1 poor prognostic factor. In the high-risk group, 459 (62.2%) patients received AC. The adjuvant treatments were as follows: a semi-monthly regimen of 5-FU and leucovorin (LV5FU2 regimen, *n* = 20); capecitabine (*n* = 119); a semi-monthly regimen of 5-FU, leucovorin, and oxaliplatin (FOLFOX regimen, *n* = 132); and a regimen of capecitabine and oxaliplatin (*n* = 189).

### Determination of LC cutoff value

**Figure 3:**
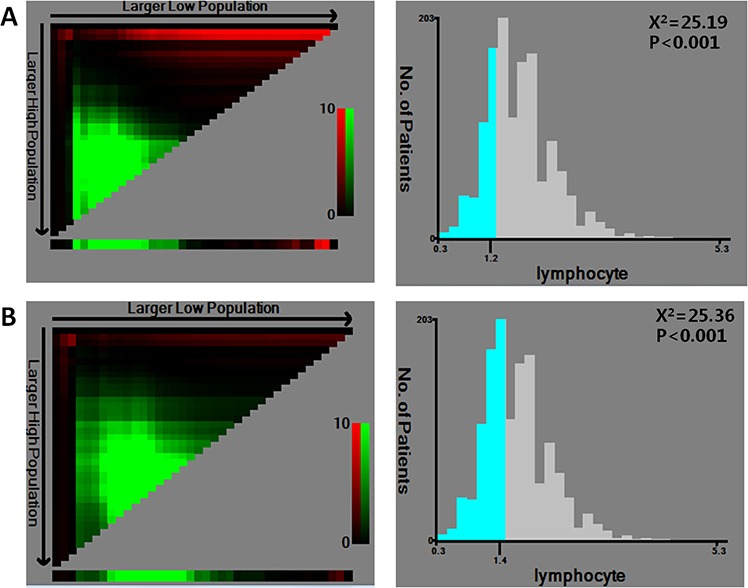
X-tile analysis of survival data within the cohort X-tile plots of training sets are shown in the left panels. The plot shows the chi-square log-rank values created when the cohort was divided into two groups. The optimal cutoff point highlighted by the black circle in the left panels is shown on a histogram of the entire cohort (right panels). *P* values were determined using the cutoff point defined in the training subset to parse a separate validation subset. **A.** The optimal cutoff point for OS (LC = 1.2, x2 = 25.19, *p* < 0.001). **B.** The optimal cutoff point for DFS (LC = 1.4, x2 = 25.36, *p* < 0.001).

To analyze the predictive value of LC for DFS and OS in patients with stage II CRC, X-tile software was used. This software allowed us to define an optimal cutoff point that defined the LC value required to predict prognosis. For OS, the maximum of ×2 log-rank values of 25.19 (*p* < 0.001) was achieved when applying an LC of 1.2 as the cutoff value (Fig. 3A), but for DFS, the maximum log-rank statistical value was 25.36 (*p* < 0.001) when the cutoff value was 1.4 (Fig. 3B). Therefore, we utilized a median of 1.3 as the optimal cutoff value for both OS and DFS in stage II CRC.

### Correlation of LC with clinicopathological characteristics

Patient baseline characteristics are shown in Table 1. The incidence of low LC was 28.1% (373/1332). As a continuous variable, low LC correlated with tumor location (*p* = 0.012), T stage (*p* = 0.025) and high risk (*p* = 0.034). As a dichotomous variable, low LC was associated with vessel invasion (*p* = 0.048).

**Table 1:** Comparison of baseline clinical characteristics based on LC

Factors	N	Lymphocyte count	*p* -Value	Lymphocyte count		*p* Value
		Mean ± SD		≤1.3	>1.3	
Age			0.173			0.146
<60	411	1.73 ± 0.57		103 (27.81%)	308 (32.05%)	
≥60	921	1.65 ± 0.63		270 (72.19%)	651 (67.85%)	
Sex			0.957			0.117
Male	821	1.71 ± 0.57		217 (58.18%)	604 (62.98%)	
Female	511	1.71 ± 0.61		156 (41.82%)	355 (37.02%)	
Location			0.012			0.179
Rectum	644	1.75 ± 0.74		169 (45.31%)	475 (49.53%)	
Colon	688	1.67 ± 0.61		204 (54.69%)	484 (50.47%)	
Tumor size (cm)			0.116			0.200
≤5.0	952	1.73 ± 0.59		257 (68.90%)	695 (72.47%)	
>5.0	380	1.67 ± 0.59		116 (31.10%)	264 (27.53%)	
T stage			0.025			0.2317
T3	902	1.74 ± 0.59		242 (64.87%)	660 (68.82%)	
T4	430	1.66 ± 0.57		131 (35.13%)	299 (31.18%)	
Vessel invasion			0.076			0.048
Negative	1232	1.72 ± 0.59		336 (90.01%)	896 (93.43%)	
Positive	100	1.61 ± 0.60		37 (9.99%)	63 (6.57%)	
Lymph node sampling			0.152			0.105
<12	201	1.66 ± 0.59		66 (17.69%)	135 (14.08%)	
≥12	1131	1.72 ± 0.59		307 (82.31%)	824 (85.92%)	
Perineural invasion			0.501			0.456
Negative	1171	1.72 ± 0.59		324 (86.86%)	847 (88.32%)	
Positive	161	1.68 ± 0.59		49 (13.14%)	112 (11.68%)	
Grade			0.493			0.365
Well	242	1.75 ± 0.58		59 (15.78%)	183 (19.10%)	
Moderate	988	1.71 ± 0.59		285 (76.47%)	703 (73.28%)	
Poor	102	1.69 ± 0.57		29 (7.75%)	73 (7.62%)	
CEA			0.264			0.056
≤5.0	800	1.72 ± 0.57		213 (60.34%)	587 (66.25%)	
>5.0	439	1.68 ± 0.60		140 (39.66%)	299 (33.75%)	
Risk factor			0.034			0.075
No	594	1.75 ± 0.60		152 (40.75%)	442 (46.09%)	
Yes	738	1.68 ± 0.58		221 (59.25%)	517 (53.91%)	

### Clinical outcome of LC status or LC status combined with high-risk factors in stage II colorectal cancer

**Figure 4:**
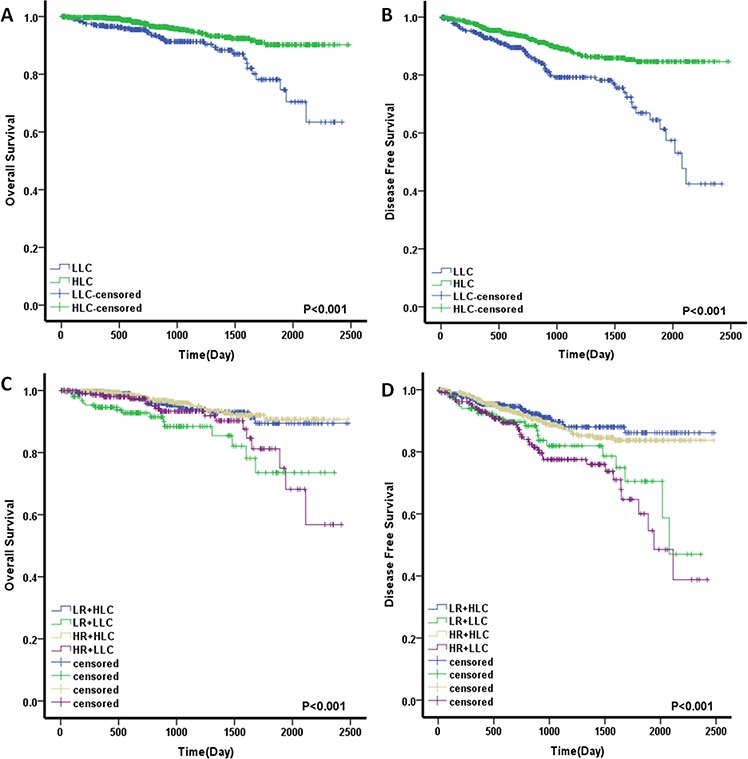
Prognostic significance of LC and LC combined with high-risk factors for stage II colorectal cancer patients assessed by Kaplan-Meier analyses **A.** A low LC value was significantly associated with a worse OS. **B.** A low LC value was significantly associated with a worse DFS. **C.** LC combined with high-risk factors for OS. **D.** LC combined with high-risk factors for DFS. The four subgroups were divided according to combinations of LC status and high-risk factors. Low LC, LLC; High LC, HLC; High risk, HR; Low risk, LR.

In Kaplan-Meier analyses, patients with low LCs exhibited decreased postoperative OS and shorter DFS (Fig. 4A, 4B). The 5-year OS and DFS rates of low-LC patients were 74.6% and 61.3%, respectively, which were significantly lower than those of high-LC patients (OS, 90.2%; DFS, 84.6%; *p* < 0.001). We divided the patients into low- and high-risk groups. Patients with high LC values and a low-risk status had the best prognosis, whereas those with low LC values and a high-risk status had the worst prognosis, with the lowest OS and DFS (*p* < 0.001) (Fig. 4C, 4D). Univariate analysis showed that poor DFS was significantly associated with perineural invasion (HR, 2.140; *p* < 0.001), lymph node sampling <12 (HR, 1.618; *p* = 0.011), CEA > 5.0 μg/ml (HR, 1.682, *p* = 0.002) and LC ≤ 1.3 (HR, 2.214; *p* < 0.001). Furthermore, LC ≤ 1.3 was also associated with poor OS (HR, 2.486; *p* < 0.001) (Table 2). Multivariable analysis of survival revealed that perineural invasion (HR, 1.957; 95% CI, 1.292–2.965; *p* = 0.002), CEA > 5.0 μg/ml (HR, 1.488, *p* = 0.022) and LC (HR, 2.090; 95% CI, 1.493–2.925; *p* < 0.001) were independent prognostic factors for DFS. Additionally, LC had a significant effect on OS (HR, 2.425; 95% CI 1.507–3.901; *p* < 0.001). In the multivariable model, the combination of LC and risk factors was also confirmed to be an independent prognostic indicator, especially for DFS (*p* < 0.001) (Table 3).

**Table 2:** Univariate analyses of factors associated with overall survival and disease-free survival

Variable	Disease-free survival		Overall survival	
	Hazard ratio (95% CI)	*p* Value	Hazard ratio (95% CI)	*p* Value
Sex (female vs. male)	1.192 (0.864–1.644)	0.284	1.021 (0.640–1.629)	0.930
Age, y (<60 vs. ≥60)	0.791 (0.551–1.135)	0.203	0.500 (0.240–1.043)	0.064
Location (colon vs. rectum)	0.939 (0.683–1.291)	0.700	0.825 (0.522–1.305)	0.411
Tumor size (cm; ≤5 vs. >5)	0.964 (0.675–1.375)	0.838	0.778 (0.480–1.259)	0.307
T stage (T4 vs. T3)	1.365 (0.985–1.891)	0.061	1.454 (0.913–2.315)	0.115
Vessel invasion (positive vs. negative)	1.413 (0.853–2.340)	0.179	1.448 (0.721–2.909)	0.298
Perineural invasion (positive vs. negative)	2.140 (1.448–3.164)	<0.001	1.685 (0.925–3.070)	0.088
Lymph node sampling (<12 vs. ≥12)	1.618 (1.118–2.342)	0.011	1.156 (0.661–2.020)	0.611
Tumor differentiation (III vs. I-II)	1.013 (0.723–1.418)	0.941	1.399 (0.869–2.251)	0.167
CEA (μg/ml; >5.0 vs ≤5.0)	1.682 (1.203–2.352)	0.002	2.107 (1.317–3.371)	0.002
LC (≤1.3 vs. >1.3)	2.214 (1.607–3.051)	<0.001	2.486 (1.575–3.926)	<0.001
Combination of LC and risk factor				
I versus II	0.444 (0.297–0.664)	<0.001	0.521 (0.269–1.012)	0.054
I versus III	0.809 (0.491–1.333)	0.406	1.520 (0.783–2.952)	0.216
I versus IV	0.378 (0.239–0.597)	<0.001	0.456 (0.244–0.851)	0.014

NOTE: Univariate analysis, Cox proportional hazards regression model. I, high LC /low risk; II, low LC/low risk; III, high LC/high risk; IV, low LC/high risk. Abbreviation: 95% CI, 95% confidence interval

**Table 3:** Multivariable analyses of factors associated with overall survival and disease-free survival

Variable	Disease-free survival		Overall survival	
	Hazard ratio (95% CI)	*p* Value	Hazard ratio (95% CI)	*p* Value
Age, y (<60 vs. ≥60)	0.845 (0.542–1.236)	0.365	0.546 (0.266–1.116)	0.095
T stage (T4 vs. T3)	1.217 (0.859–1.725)	0.269	1.228 (0.749–2.011)	0.415
Vessel invasion (positive vs. negative)	1.168 (0.656–1.890)	0.438	1.125 (0.535–2.365)	0.755
Perineural invasion (positive vs. negative)	1.957 (1.292–2.965)	0.002	1.527 (0.812–2.871)	0.189
Lymph node sampling (<12 vs. ≥12)	1.460 (0.986–2.163)	0.059	1.084 (0.605–1.941)	0.786
CEA (μg/ml; >5.0 vs ≤5.0)	1.488 (1.059–2.091)	0.022	1.794 (1.112–2.893)	0.017
LC (≤1.3 vs. >1.3)	2.090 (1.493–2.925)	<0.001	2.425 (1.507–3.901)	<0.001
Combination of LC and risk factor				
I versus II	0.485 (0.297–0.799)	0.004	0.626 (0.328–1.265)	0.215
I versus III	0.966 (0.585–1.632)	0.895	1.735 (0.905–3.370)	0.116
I versus IV	0.465 (0.308–0.720)	<0.001	0.488 (0.252–0.910)	0.025

NOTE: Multivariable analysis, Cox proportional hazards regression model. I, high LC /low risk; II, low LC/low risk; III, high LC/high risk; IV, low LC/high risk. Abbreviation: 95% CI, 95% confidence interval

### Predictive value of LC for the use of adjuvant chemotherapy in high-risk patients

**Figure 5:**
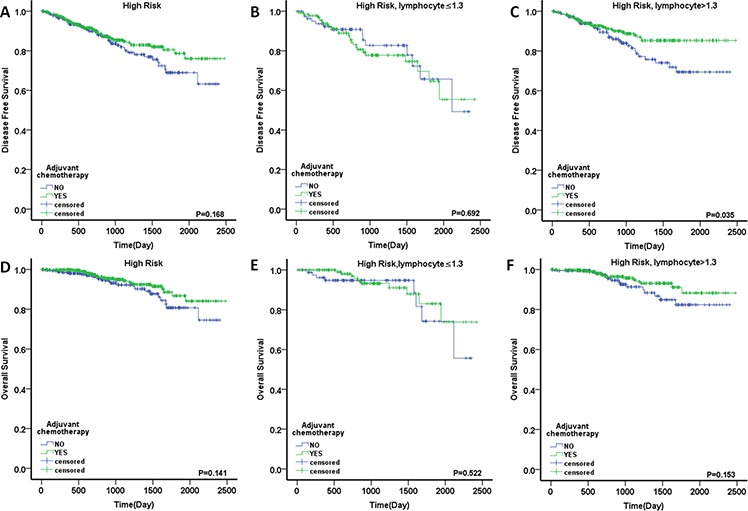
Outcome of adjuvant chemotherapy in stage II colorectal cancer DFS in the patients with **A.** high risk, **B.** high risk and LC ≤1.3, **C.** high risk and LC >1.3; OS in the patients with **D.** high risk, **E.** high risk and LC ≤1.3 and **F.** high risk and LC >1.3.

**Figure 6:**
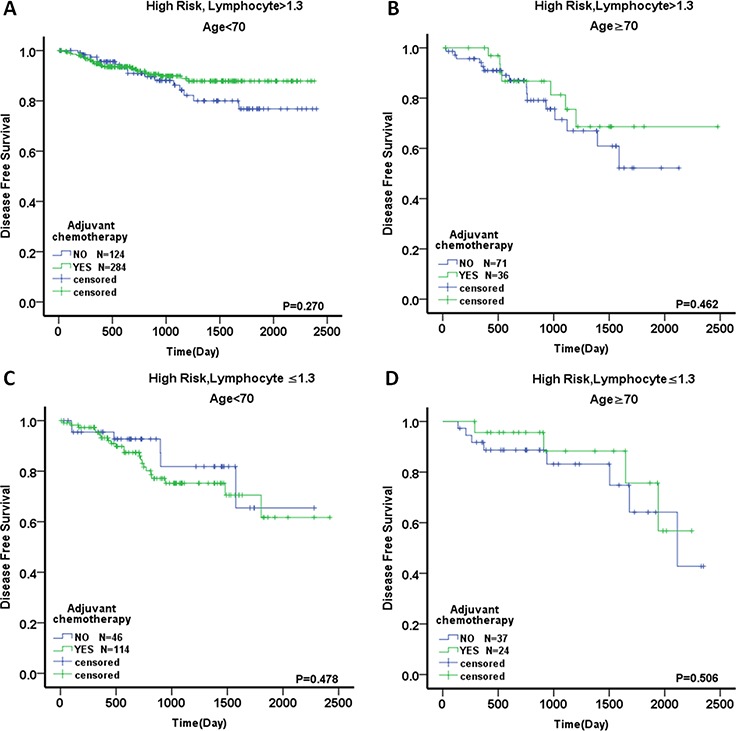
Outcome of adjuvant chemotherapy in stage II colorectal cancer divided by age DFS in the patients with **A.** high risk, LC >1.3 and age <70, **B.** high risk, LC >1.3 and age ≥70, **C.** high risk, LC ≤1.3 and age <70, and **D.** high risk, LC ≤1.3 and age ≥70.

The 738 high-risk patients were divided into two groups: a low-LC (*n* = 223, 30.2%) and a high-LC group (*n* = 515, 69.8%). Among the two groups, 136 patients (61.0%) and 323 patients (62.7%) received AC. The baseline characteristics are described in Table 4. In the Kaplan–Meier analyses, the patients treated with AC exhibited a nonsignificant improvement in postoperative DFS and OS compared with untreated patients (DFS, *p* = 0.168; OS, *p* = 0.141; Fig. 5A, 5D). Interestingly, in the high-LC group, the patients treated with AC had a significant DFS advantage compared with those who did not receive AC (5-year DFS rate of 85.3% vs. 69.4%; HR, 0.594; 95% CI, 0.364–0.970; *p* = 0.035; Fig. 5C). There was also a nonsignificant OS benefit for the AC group (88.2% vs. 82.4%; HR, 0.591; 95% CI, 0.285–1.225; *p* = 0.153; Fig. 5F). It is notable that there were no differences in either DFS or OS in the low-LC patients regardless of receipt of AC (for DFS: HR, 1.141; 95% CI, 0.594–2.189; *p* = 0.692; for OS: HR, 0.738; 95% CI, 0.290–1.876; *p* = 0.522; Fig. 5B, 5E). In order to exclude potentially confounding factors, we compared the baseline of two groups. No obvious differences were noted in sex, tumor location and risk factors except for age between the patients received AC or not. Given the recent evidence suggesting that the benefits of AC might differ based on age [8], we further explored for effect modification by dichotomizing the cohort into elderly patients (aged ≥70 years) and younger patients (aged <70 years). In the high-LC group, there was a nonsignificant DFS advantage in both younger and elderly patients treated with AC (for younger patients: HR, 0.704; 95% CI, 0.375–1.318; *p* = 0.273; for elderly patients: HR, 0.717; 95% CI, 0.294–1.748; *p* = 0.464; Fig. 6A, 6B). However, no obvious DFS improvement was found in the low-LC group, especially for the younger patients (for younger patients: HR, 1.386; 95% CI, 0.561–3.426; *p* = 0.480; for elderly patients: HR, 0.665; 95% CI, 0.198–2.233; *p* = 0.509; Fig. 6C, 6D). Next, we evaluated individual factors associated with poor prognosis in the high-risk patients. The patients with T4 tumors demonstrated significant improvements in DFS following the use of AC both in the high-risk group and the group with high LC. (HR, 0.525; 95% CI, 0.315–0.876; *p* = 0.014; and HR, 0.442; 95% CI, 0.227–0.860; *p* = 0.013, respectively; Table 5).

**Table 4:** Comparison of baseline clinical characteristics in high-risk patients divided by LC status

Variable	LC ≤1.3 Adjuvant chemotherapy		*p*	LC >1.3 Adjuvant chemotherapy		*P*
	YES	NO		YES	NO	
Mean age, years	59.71 ± 1.02	66.02 ± 1.36	<0.001	57.48 ± 0.61	63.05 ± 0.92	<0.001
Age ≥70 (%)	15.65	41.53		13.73	38.13	
Sex			0.675			0.636
Male	76 (55.88%)	51 (58.62%)		202 (62.54%)	121 (63.02%)	
Female	60 (44.12%)	36 (41.38%)		121 (37.46%)	71 (36.98%)	
Location			0.881			0.702
Colon	93 (68.38%)	59 (67.82%)		207 (64.09%)	120 (62.50%)	
Rectum	43 (31.62%)	28 (32.18%)		116 (35.91%)	72 (37.50%)	
Risk factor			0.994			0.603
Single	98 (72.06%)	61 (70.11%)		248 (76.78%)	142 (73.96%)	
Two	31 (22.79%)	20 (22.99%)		63 (19.50%)	44 (22.92%)	
Three or more	7 (5.15%)	6 (6.90%)		12 (3.72%)	6 (3.12%)	
Overall	136	87		323	192	

**Table 5:** Univariate analysis of the effect of adjuvant chemotherapy on outcomes, stratified by specific risk subgroups

Variable	Disease-free survival		Overall survival	
	Hazard ratio (95% CI)	*p* Value	Hazard ratio (95% CI)	*p* Value
**High risk (*n* = 738)**	0.755 (0.513–1.113)	0.156	0.645 (0.364–1.143)	0.133
Poorly differentiated tumor (*n* = 156)	0.670 (0.305–1.471)	0.319	0.418 (0.122–1.433)	0.165
Vessel invasion positive (*n* = 96)	0.675 (0.248–1.831)	0.440	0.989 (0.263–3.719)	0.988
Perineural invasion positive (*n* = 159)	0.757 (0.375–1.527)	0.437	0.772 (0.258–2.306)	0.643
Lymph nodes sampled <12 (*n* = 201)	0.787 (0.407—1.522)	0.477	0.703 (0.263–1.876)	0.481
T4 (*n* = 430)	0.525 (0.315–0.876)	0.014	0.548 (0.264–1.140)	0.107
**High risk LC >1.3 (*n* = 515)**	0.594 (0.364–0.970)	0.035	0.591 (0.285–1.225)	0.153
Poorly differentiated tumor (*n* = 113)	0.559 (0.188–1.667)	0.297	0.412 (0.069–2.465)	0.331
Vessel invasion positive (*n* = 62)	0.745 (0.210–2.646)	0.649	0.924 (0.186–4.590)	0.923
Perineural invasion positive (*n* = 110)	0.543 (0.230–1.281)	0.163	0.785 (0.210–2.933)	0.719
Lymph nodes sampled <12 (*n* = 110)	0.947 (0.409—2.193)	0.899	0.789 (0.228–2.731)	0.709
T4 (*n* = 293)	0.442 (0.227–0.860)	0.013	0.467 (0.183–1.235)	0.127

### Prognostic value of LC in high-risk patients treated with adjuvant chemotherapy

**Figure 7:**
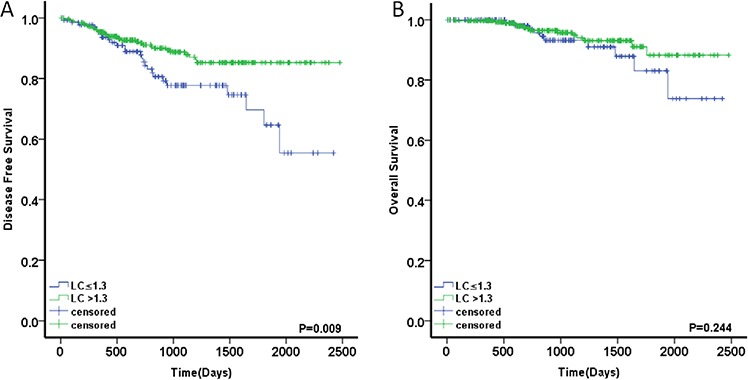
Prognostic value of LC in high-risk patients treated with adjuvant chemotherapy **A.** DFS in the patients with LC ≤1.3 or >1.3 **B.** OS in the patients with LC ≤1.3 or >1.3.

To evaluate the prognostic value of LC status in high-risk patients treated with AC,we used Kaplan–Meier to estimate the DFS and OS. Patients with high LC had a significant DFS advantage compared with those with low LC(3-year DFS rate of 88.7% vs. 77.7%6-year DFS rate of 85.3% vs 55.4%) (Fig. 7A, 7B). In univariate and multivariate Cox regression models, Low LC was significantly associated with poorer DFS (HR, 1.978; 95% CI, 1.178–3.319; *p* = 0.010). Other DFS prognostic variables are presented in Table 6 and include perineural invasion (*p* = 0.038), number of lymph nodes sampled (*p* = 0.013) and additional risk factors (*p* = 0.018). Only the patients with two or more poor prognostic factors were associated with a worse OS (*p* = 0.037). The chemotherapy regimen was not found to be associated with either DFS or OS. In the multivariate analyses, low LC (HR, 1.885; 95% CI, 1.112–3.196; *p* = 0.019), perineural invasion (HR, 1.965; 95% CI, 1.024–3.770; *p* = 0.042) and number of lymph nodes sampled (HR, 1.964; 95% CI, 1.105–3.493; *p* = 0.021) were independent prognostic factors for DFS. Additional risk factors were found to be independently associated with OS, with HR = 2.290, 95% CI = 1.015–5.167, and *p* = 0.046 (Table 6).

**Table 6:** Univariate and multivariable analyses of factors associated with overall survival and disease-free survival of high-risk patients treated with adjuvant chemotherapy

Variable	Disease-free survival		Overall survival	
	Hazard ratio (95% CI)	*p* Value	Hazard ratio (95% CI)	*p* Value
**Univariate Analyses**				
Sex (female vs. male)	1.198 (0.715–2.007)	0.493	1.295 (0.581–2.883)	0.527
Age, y (<70vs. ≥70)	0.714 (0.370–1.378)	0.316	0.417 (0.173–1.007)	0.052
Location (colon vs. rectum)	0.856 (0.582–1.171)	0.715	0.765 (0.317–1.845)	0.550
Tumor size (cm; ≤5 vs. >5)	1.302 (0.702–2.413)	0.402	0.707 (0.303–1.652)	0.423
T stage (T4 vs. T3)	1.430 (0.854–2.394)	0.174	1.213 (0.529–2.778)	0.648
Vessel invasion (positive vs. negative)	1.704 (0.884–3.286)	0.112	1.754 (0.655–4.703)	0.264
Perineural invasion (positive vs. negative)	1.808 (1.035–3.160)	0.038	1.784 (0.737–4.319)	0.199
Number of lymph nodes (<12 vs. ≥12)	1.952 (1.154–3.301)	0.013	1.068 (0.452–2.526)	0.880
Tumor differentiation (III vs. I-II)	1.051 (0.557–1.987)	0.877	1.262 (0.430–3.703)	0.672
LC (≤1.3 vs. >1.3)	1.978 (1.178–3.319)	0.010	1.612 (0.716–3.631)	0.249
Risk factor (two or more vs. one)	1.915 (1.120–3.273)	0.018	2.374 (1.053–5.352)	0.037
Chemotherapy (bi-therapy vs. monotherapy)	0.873 (0.449–1.695)	0.688	0.637 (0.256–1.587)	0.333
**Multivariable Analyses**				
Age, y (<70 vs. ≥70)	0.874 (0.446–1.713)	0.695	0.436 (0.180–1.056)	0.066
Perineural invasion (positive vs. negative)	1.965 (1.024–3.770)	0.042		
Number of lymph nodes (<12 vs. ≥12)	1.964 (1.105–3.493)	0.021		
Risk factor (two or more vs. one)	1.195 (0.636–2.244)	0.579	2.290 (1.015–5.167)	0.046
LC (≤1.3 vs. >1.3)	1.885 (1.112–3.196)	0.019		

## DISCUSSION

In the present study, we performed a large-scale retrospective cohort study on patients with stage II CRC and described three major findings. First, LC is an independent poor prognostic factor for DFS and OS in stage II CRC. Second, by comparing high-risk patients who did and did not receive AC, we found that patients with low LC did not benefit from AC, whereas patients with high LC had a significant DFS advantage, especially patients with T4 stage disease. Finally, low LC was also associated with poor prognosis in high-risk patients treated with AC. To our knowledge, this is the first study to determine the predictive value of pretreatment LC in stage II CRC and high-risk patients treated with AC.

In recent years, several reports have documented a correlation between peripheral LC and survival in patients with various types of malignancies, including pancreatic ductal adenocarcinoma, esophageal squamous cell carcinoma, and lung cancer [16–18]. However, LC cutoff values vary among different cancers, ranging from 1.0 to 1.9 [14, 16–18]. Therefore, in this study, X-tile software was used to analyze the optimum cutoff value of LC for stage II CRC. The data showed that a median LC of 1.3 was the optimal cutoff value for OS and DFS, and this value was also employed in previous studies [12, 19].

Next, we evaluated the efficacy of LC for predicting postoperative survival. LC was associated with vessel invasion and high-risk factors, which is concordant with data from Jian Zhang et al [20], who found that low LC was related to lymphatic invasion in non-small cell lung cancer. Furthermore, we demonstrated that patients with low LCs had significantly decreased postoperative 5-year OS (74.6% vs. 90.2%, *p* < 0.001) and shorter DFS (61.3% vs. 84.6%, *p* < 0.001) compared to those with high LCs. In multivariate analysis, low LC was a significant and strong independent poor predictive factor for DFS (*p* < 0.001) and OS (*p* < 0.001). It is well known that the high-risk factors for stage II CRC include perforation, T4 tumors, suboptimal lymph node sampling, poor differentiation, colonic obstruction, and lymphovascular or perineural invasion [3, 5]. In the current study, we excluded the impact of perforation and colonic obstruction on baseline LC. Among the remaining features, perineural invasion (HR, 2.140; *p* < 0.001) and suboptimal lymph node sampling (HR, 1.618; *p* = 0.011) were independent prognostic factors for reduced DFS. Moreover, the combination of LC and risk factor status was also confirmed as an independent prognostic indicator, especially for DFS (*p* < 0.001), and was even stronger than LC alone.

There is substantial evidence that the systemic immune response of a host against a tumor is a vital independent prognostic factor. Solid tumors are generally infiltrated with leukocyte subsets, among which lymphocytes play a major role in the immune response by mediating the immunologic destruction of various cancers [21–23]. In CRC, Jass et al [24] and Ropponen et al [25] noted that conspicuous lymphocytic infiltration along the invasive tumor margin is an independent prognostic factor for improved survival. In most cases, these lymphocytes are either CD4+ or CD8+ T cells [26, 27]. Differentiated CD8+ T cell clusters have a pivotal role in tumor growth control via their induction of cytotoxic T-cell killing and apoptosis [28]. The quantity of CD8+ T cells that are present significantly correlates with improved disease-specific survival in CRC [29]. Meanwhile, CD4+ T cells play a central role in orchestrating the immune response to cancer [30]. Although no direct research has demonstrated that peripheral lymphocyte count correlates with the number of tumor-infiltrating lymphocytes, some studies have indicated an association between them. Romano et al [31] found that preoperative treatment with recombinant human IL-2 significantly increased total peripheral blood lymphocytes and CD4 cells, which resulted in higher lymphocyte tumor infiltration. Additionally, Chiba et al [29] observed that the prognostic impact of intraepithelial CD8+ T cells in CRC is more evident when the follow-up period is longer. Disease-specific survival curves for patients with different levels of intraepithelial CD8+ T cells are similar during the first 1–2 years of follow-up and subsequently diverge. These survival curves were very similar to the peripheral LC results in our study. Moreover, Pages et al [32] also demonstrated that early metastatic invasion is negatively associated with tumor-infiltrating immune cells, which agreed with our results showing that low peripheral LC was significantly associated with vascular invasion. These results suggest that local immune responses in tumor tissues might not actually be confined to local sites but rather may reflect systemic anti-tumor immune responses. Therefore, lymphocytopenia, as an index of a generalized depressed immune status, might adversely influence survival due to reduced systemic and local host responses to tumors.

The role of AC is well established in stage III CRC, but its benefit to stage II patients remains controversial. The MOSAIC study demonstrated no improvement in DFS or OS in 899 patients with stage II disease (DFS: HR, 0.84; *p* = 0.258; OS: HR, 1.00; *p* = 0.986). A trend toward improved outcome was noted among high-risk stage II patients (DFS: HR, 0.72; OS: HR, 0.91; *p* = 0.648) [7]. Conversely, an analysis of SEER-Medicare data demonstrated a lack of survival benefit from AC in the same population (HR, 1.03; 95% CI, 0.94–1.15; *p* = 0.47) [8]. Therefore, there is considerable interest in elucidating additional predictive biomarkers that could improve outcome through patient classification. It is notable that most prior research has focused on the factors associated with poor prognosis in stage II patients while ignoring host immune status. Ropponen et al [25] confirmed that there is an inverse correlation between the presence of tumor infiltrating lymphocytes and tumor stage in CRC: infiltrating CD8+ T cells are more prominent during early stages (stages I and II) and decrease in number during advanced stages (stages III and IV). This situation might be a consequence of systemic immune suppression in patients with advanced-stage disease. This possibility in turn suggests that immune status plays an important role in the outcome of early-stage CRC because micrometastases are more amenable to elimination via host immune response. Therefore, in contrast to advanced CRC, more attention should be paid to the combination of immune status and poor prognostic factors when evaluating treatment effect in stage II patients. In the current study, we demonstrated that high-risk patients treated with AC exhibited nonsignificant improvements in DFS and OS (DFS: *p* = 0.168, OS: *p* = 0.141). High-LC patients who received AC had a significant DFS advantage compared to those not treated with AC (*p* = 0.035; HR, 0.594). However, there was no difference or trend in DFS or OS in low-LC patients regardless of the receipt of AC (DFS: *p* = 0.692; HR, 1.141; OS: *p* = 0.522; HR, 0.738). In multivariate analysis, low LC was independently associated with poorer DFS (*p* = 0.019; HR, 1.885) in high-risk patients treated with AC. The above research supports our hypothesis that patient immune status substantially impacts the outcome of AC. Stage II CRC patients with a normal immune status might gain more benefit from adjuvant treatment than those with a poor immune status, who might derive no benefit, regardless of the presence of high-risk factors.

Some studies have suggested an association between pretreatment lymphopenia and poor cancer survival or poor response to chemotherapy or radiotherapy. Lissoni et al [21] showed that lymphocytopenia prior to chemotherapy is associated with lower treatment efficacy in terms of objective tumor regression rates in patients with metastatic solid tumors. Kitayama et al [33] and Chi Hwan Choi [34] suggested that pretreatment LC was an important determinant of preoperative radiotherapy efficacy in advanced rectal cancer. The mechanism underlying the association between low LC and decreased chemotherapy efficacy is not well understood. The possible reasons are as follows: 1) Chemotherapy might cause lymphocyte depletion and alter the balance of lymphocyte subpopulations [35], which might further decrease LC in patients with lymphocytopenia. Lymphocyte depletion, especially of T cells, potentially compromises the effectiveness of the anti-tumor immune response. 2) Lymphopenia represents an ineffective anti-tumor immune response against cancer cells/tissues, which leads to tumor recurrence regardless of the receipt of AC. 3) Lymphopenia appears to lead to severe chemotherapy-induced hematological toxicity [13], which results in a significant disadvantage for patients. Collectively, our results suggest that in stage II CRC, AC should be used in high-risk patients with a favorable immune status (LC > 1.3), especially those with stage T4 disease. For high-risk patients with a poor immune status (LC ≤ 1.3), AC should not be recommended because it does not benefit survival and potentially leads to severe toxicity.

There are several limitations to the current study. First, although we adopted rigorous inclusion and exclusion criteria, it has been shown that diabetes mellitus and renal and/or hepatic failure might potentially affect lymphocytes [36, 37]. Additionally, anti-diabetic drugs, anti-hypertensive drugs, and/or other medications might potentially affect LC. Therefore, larger prospective studies are needed to confirm these preliminary results. Second, toxicity information is not routinely collected in our database, thereby limiting our ability to explore this aspect in greater detail. Third, different schedules of chemotherapy were used, leading to potential differences in the outcome of AC. However, these limitations should be viewed within the context of the study's strengths, including its population-based nature, generalizability, and relatively large sample of patients with stage II CRC.

In conclusion, our study is the first to demonstrate that pretreatment LC is an independent prognostic factor for survival in stage II CRC patients. Furthermore, pretreatment LC is also an independent prognostic factor for high-risk patients treated with AC. Most importantly, pretreatment LC reliably predicts chemotherapy efficacy in high-risk patients. Therefore, based on its easy attainability, pretreatment LC might be used to select patients with high-risk disease, evaluate intensified therapy, and develop novel agents to improve patient outcome.

## PATIENTS AND METHODS

Clinical data were collected from Fudan University Shanghai Cancer Center (FDSCC), Shanghai, China. The FDSCC dataset was built prospectively and included records of all colorectal cancer patients treated at FDSCC since January, 2006. A total of 1494 patients were retrieved from the database. Patients with the following inclusion criteria were enrolled: 1) hospitalized for primary diagnosis and therapy; 2) CRC confirmed by histopathology with curative primary tumor resection and staged according to TNM criteria (AJCC criteria 2009); 3) stage II disease; 4) preoperative blood test results obtained within 1 week prior to surgery; and 5) all clinical data were available. Patients with the following criteria were excluded: 1) incomplete resection with microscopic or macroscopic residual tumors; 2) previous or concomitant other cancers; 3) absence of detailed information or clinical data; 4) clinical evidence of infection, other inflammation, or hematologic disease, or use of hematology-influencing drugs within one month; 5) preoperative neoadjuvant therapy; 6) complete intestinal obstruction or perforation; and 6) contact lost during follow-up.

The following data were collected from the medical records of each patient: age (<60 and ≥60 years); sex (male or female); tumor size (≤5 and >5 cm); tumor location (colon and rectum); T stage (T3 and T4); differentiation (well, moderate, and poor); vessel invasion (negative and positive); perineural invasion (negative and positive); and number of lymph nodes sampled (<12 and ≥12). Vessel and perineural invasion was recorded according to the results of pathologic reports. The 7th edition was used to define the T stage as follows: T3, invasion of the adventitia; T4, invasion of adjacent structures. As part of the physical examinations, peripheral blood was collected before surgery, and peripheral lymphocytes were counted using an automated hematology analyzer (Sysmex XE-5000; Sysmex, Kobe, Japan).

LC cutoff points were produced and analyzed using the X-tile program (http://www.tissuearray.org/rimmlab/), which identified the cutoff with the minimum *p* values from log-rank ×2 statistics in terms of survival [15]. The endpoints assessed were disease-free survival (DFS) and overall survival (OS). For assessment of DFS, recurrence was defined as time from operation to development of local, nodal (regional) and distant metastasis. OS was defined as time from operation to date of death.

Patients with stage II disease complicated by T4 tumors, suboptimal lymph node sampling (<12 lymph nodes), the presence of lymphovascular or perineural invasion or poor differentiation were categorized as “high-risk”. For the high-risk group, the role of postoperative AC is controversial, and adjuvant therapy was not mandatory. The most frequent ACs included 5-fluorouracil (5-FU) and oxaliplatin. In all cases, treatment regimens were based on recommended dosing ranges and schedules. The duration of AC was 6 months.

Independent t tests were used to compare the LC as a continuous variable. Chi-square tests were used to determine the significance of differences for patients grouped by LC as a dichotomous variable. Survival curves were generated using Kaplan–Meier estimates, and differences between the curves were analyzed by a log-rank test. Cox regression models were built for analysis of risk factors for survival outcomes in CRC patients. Continuous variables, reported as LC, were compared using the Wilcoxon rank-sum test. Multivariate analyses with a Cox proportional hazards model were used to test independence, significance, and hazard discrimination. Covariates included in the model are given in the result tables as previously reported. Statistical analyses were performed using the statistical software package SPSS for Windows, version 19 (SPSS Inc., Chicago, IL, USA). A two-tailed *p* value < 0.05 was considered significant.
